# Using Complexity to Simplify Knowledge Translation: Comment on "Using Complexity and Network Concepts to Inform Healthcare Knowledge Translation"

**DOI:** 10.15171/ijhpm.2017.139

**Published:** 2017-12-31

**Authors:** Anita Kothari, Shannon L. Sibbald

**Affiliations:** School of Health Studies, Faculty of Health Sciences, Western University, London, ON, Canada.

**Keywords:** Knowledge Translation (KT), Evidence-Based Practice, Implementation Science, Complex Adaptive Systems, Networks, Complexity

## Abstract

Putting health theories, research and knowledge into practice is a challenge referred to as the knowledge-toaction gap. Knowledge translation (KT), and its related concepts of knowledge mobilization, implementation science and research impact, emerged to mitigate this gap. While the social interaction view of KT has gained currency, scholars have not easily made a link between KT and the concept of complexity. Kitson and colleagues suggest we ought to examine the role of complexity in KT processes using defined theories and concepts borrowed from network and complex adaptive systems theory. They further argue that better KT outcomes might be achieved using this new lens. There remain, however, several critical considerations for this sort of theory application to work in the real-world. Complexity and network theory offer explanatory power about the KT problem, but these theories are less helpful for understanding solutions.

The article *Using Complexity and Network Concepts to Inform Healthcare Knowledge Translation*^1^ was based on the development of a knowledge translation (KT) strategic framework in a Faculty of Health and Medical Sciences for an Australian university. The fact that several researchers and their diverse research programs were considered, as was the complexity literature across many fields, adds to the strength of the arguments in the paper. The authors propose that a reconceptualization of how we think about KT is needed to move the practice of KT forward in healthcare. Kitson et al suggest, as have others, that the idea of KT as a linear push-pull process has been unsuccessful. As a result, they note that the field has focused on more dynamic representations of KT. There is greater attention to KT as a social, iterative phenomenon with attention to the interaction between people. Kitson et al put forth an even broader view of KT using a complexity lens to incorporate relationships, organizations, and politics. This view also notes the interdependency of sub-systems, illustrated by different research teams working on problem identification, knowledge creation, knowledge synthesis, implementation, and evaluation. The authors argue that for knowledge to be created, taken up successfully and have meaningful and sustained impact, the community, health, government, education and research sectors need to be closely connected. They call the sub-systems, the sectors and the interdependency the “KT Complexity Network.” Drawing on network and complex adaptive systems theory, the paper discusses mobilizing knowledge across these sub-systems via interactions between teams and individuals. The purpose is to incorporate the larger end goal of knowledge uptake right from the start of the research process.

Complex systems or network theories “model systems and/or subsystems, in order to identify potential points for intervention or change.”^2^ In doing so these models can also help researchers and practitioners think about intended and unintended consequences of such change. Considerations of context in any KT or implementation process is essential, and considerations for broader and related contexts may be equally important. From this perspective, the process of KT needs to deal with sub-systems that are self-adapting and unpredictable, where small events can produce emerging, large outcomes which in turn cause effects elsewhere (eg, a nonlinear feedback loop). It is these considerations that KT science and practice has been lacking, and which Kitson et al attempt to fill.

There are many strengths to this article. It summarizes the current field of play around KT research as the authors describe different positions ascribed to KT research streams. It becomes clear that while there are similarities and overlap, there remains uncertainly about how to effectively practice KT. The authors introduce the nomenclature of network theory, providing just enough to give the reader a flavour for the field. They spend time discussing the opportunities these theories might present to KT in healthcare, concluding that it is both appropriate and feasible to use complexity and network theories in KT work. We agree with the authors that developing a firm understanding of complexity in healthcare might support improved KT and allow us to better support and create enabling environments.

Further discussion on some points are warranted. Kitson et al speak about the potential difficulties of engagement, and the need for leaders who will support researchers to be co-located in various sub-systems or part of a KT Team across sub-systems of the KT Complexity Network. The authors suggest appropriate reward structures to motivate researchers to participate. However, closely tied to engagement and incentives are the issues of politics and political environments, which are so important given this expanded, networked view of KT involving considerably more individuals and organizations than traditional single-team based approaches. Langley and Denis^3^ note the importance of understanding user groups’ interests, values and power relationships when trying to introduce wide-level change. Offers of rewards need to consider that individuals are positioned in a larger system characterized by its own pattern of sense-making, coalition building and rhetorical strategies.^4^ In addition to these collective processes, the research findings themselves will produce a reaction in the collective context based on opinions and preferences. If there is little dispute among an organization or research team about conceptualizations of the problem, the importance of the problem and how potential solutions ought to be evaluated, then the context is said to have low issue polarization,^4^ and research findings have a higher likelihood of being taken up. Thus, generating scientifically rigorous and useful research is not sufficient,^4,5^ as the “social determinants of action” need to be considered in each sub-system if the whole network is to be successful.

The feasibility and resources of a KT Complexity Network also warrant deeper discussion. At a minimum, the infrastructure required for such an ambitious plan would be significant, and in a zero-sum funding environment, would require reallocation of resources from things like providing patient care and supporting research studies. While acknowledging the benefits of such a network, its champions might also examine the consequences (time, attention) that are not equally distributed across the sub-networks.^3^ Understanding where the winning and losing occur – the cost-sharing equilibrium – might be important in building support across organizational and professional boundaries.^4^ Sub-systems that win more than others may also represent entry points for further interventions to support the KT Complexity Network, such as the development of the overarching KT Team as put forth by Kitson et al. Then there is the crucial element of time. As each sub-network (eg, problem identification, knowledge synthesis) executes its own program of research, it will take years for meaningful findings to emerge such that they can be taken up by the next stage of the lab-to-bedside cycle. Keeping investigators and sectors engaged over the lifespan of research processes requires further discussion.

Real world application uncovers some additional, practical challenges. First, and perhaps most importantly, how will we measure success of the KT Complexity Network? While there are a range of tools to represent and analyze networks, it is difficult to use complexity theory in outcome evaluation.^6^ Complexity theory helps us explain variation and perhaps the implicit role of motives, values and relationships in a system. Many have characterized this as organizational culture, found to be both a barrier (when culture is low) and a facilitator (as in the case of learning organizations) to implementing change.^7^ And while complexity and network theory can help us account for the nuanced levers and barriers to change, at a practical level, we are still left with very little guidance around assessment of success. The specific challenge is that “The trajectories of complex systems have histories that are a mixture of ‘much the same’ and change. For much of the time complex systems remain the same sort of thing. There are changes in them over time but these do not constitute changes of kind.”^8^ Byrne argues that system transformations like the one suggested by Kitson et al are characterized by changes in quality rather than changes in quantity.^8^ The ‘complexity of complexity theory’ is important to consider before we support its application; as an intricate science, it may not have impact in real-world decision making. Perhaps complexity is better positioned and explained as a frame of reference for understanding how KT operates.

Another challenge with building a KT Complexity Network is determining a manageable scope of the network. At first glance, it seems that limiting a network by condition, such as diabetes, might be feasible. Within health, however, there is strong recognition that risk factors are interconnected, and growing awareness that diseases and disease states are too. Further, calls for stronger connections between health and other sectors, like ecology (see https://ecohealth.net/en/ for the international association of Ecohealth) cannot be ignored. Then there is the complicated task of identifying the relevant community, health, government, and education sub-groups. The KT Complexity Network presents an intractable paradox: by considering all contexts, the Network becomes unmanageable. In short, there are practical limitations to identifying a KT Complexity Network. The recent emergence of embedded practitioner/researcher implementation roles within large healthcare systems may provide some direction about how to achieve a manageable KT complexity network. Such structures facilitate partnerships with multiple stakeholders, eg, patients, clinical researchers, practitioners, staff and administrators, but a coordinated engagement effort through the application of a KT Complexity Network might provide operational insights as well as meaningful ways to define success.

We applaud the authors for stretching our thinking and attempting to bring order to current KT theory. The principles of complexity theory provide KT researchers with a novel way of addressing the challenges associated with successful KT, eg, by drawing more attention to interconnected networks between and among sectors. It also provides an explanatory framework with which we can deepen our understanding around barriers and facilitators. As we support the continued growth around the practice of KT, we concede that complexity and network theory might be used as theories to understand the KT problem, but they are less helpful as theories supporting associated solutions, like the KT Complexity Network. We are also not convinced that a complete transformation of the conceptualization of KT is needed. Let’s not lose sight of other, established concepts related to organizational decision-making, like learning organizations or absorptive capacity, that might demonstrate the same explanatory power as complexity theory but are able to provide better solutions.

## Ethical issues

Not applicable.

## Competing interests

Authors declare that they have no competing interests.

## Authors’ contributions

Both authors contributed equally to this work. The submitted manuscript has been read and approved by both authors.
